# Early gadolinium enhancement for the detection of myocardial oedema (EGE vs T2-STIR vs ACUT2E): a new method to assess the area at risk?

**DOI:** 10.1186/1532-429X-14-S1-P31

**Published:** 2012-02-01

**Authors:** Elisa McAlindon, Jessica Harris, Andreas Baumbach, Julian W Strange, Chiara Bucciarelli-Ducci

**Affiliations:** 1 CMR Unit, NIHR BRU, Bristol Heart Institute, Bristol, UK; 2 Clinical Trials and Evaluation Unit, NIHR BRU, Bristol Heart Institute, Bristol, UK

## Background

The “gold standard” CMR sequence for assessing the myocardial oedema or area at risk following an acute coronary syndrome is controversial. Short Tau Inversion Recovery (T2-STIR) is in widespread clinical use. Steady state free precession oedema imaging (SSFP/ ACUT2E) has emerging data to support it as a more reproducible method for area at risk (AAR) assessment. More recently, early gadolinium (EGE) has been suggested as an alternative way of measuring AAR.

## Methods

30 slices in 10 patients day 2-4 following acute myocardial infarction were analysed by 3 sequences (T2-STIR, ACUT2E, and EGE). The area of oedema was planimetered and expressed as a % of slice total area. The window setting was defined as the sum of the mean signal intensity (SI) of the unaffected area plus 2 standard deviation (SD) for this area. The level setting was set at the mean SI of the unaffected area (a method used in previous studies of this type). Inter-method and inter-observer variability was assessed using the Bland Altman method. Qualitative inter-observer, and inter-method variability was assessed: each slice split into segments according to the 17 segment model and oedema in each segment scored as present of absent.

## Results

**Figure 1:**
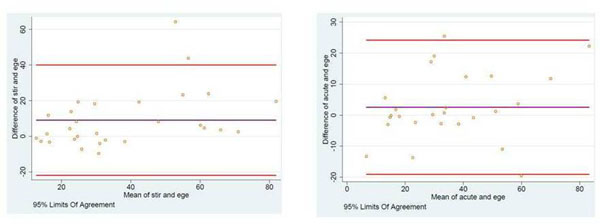
Bland Altman plots for AAR assessed by early gadolinium enhancement (ege) vs STIR (left panel) and SSFP (acute) (right panel).

The Bland Altman plots for T2-STIR vs EGE, and ACUT2E vs EGE are shown in Figure 1, demonstrating a good agreement between methods.

On qualitative assessment, there is good agreement between T2-STIR and EGE (kappa 0.73, 87% segments agree) and ACUT2E and EGE (kappa 0.72, 87% segments agree). The two established methods of assessing AAR (T2-STIR and ACUT2E) also showed good agreement, kappa 0.78, with 89% segments agreed.

On assessing qualitative inter-observer reproducibility there is a good agreement between the two observers using all 3 sequences, although SSFP appears to have the strongest interobserver agreement (T2-STIR kappa 0.56, ACUT2E kappa 0.67, EGE kappa 0.60).

## Conclusions

There is good agreement between EGE and the established methods of assessing AAR (T2-STIR and ACUT2E). EGE may offer a new method for assessing the area at risk but this needs to be further assessed in a larger patient population.

## Funding

NIHR Cardiovascular BRU, Bristol Heart Institute.

**Figure 2:**
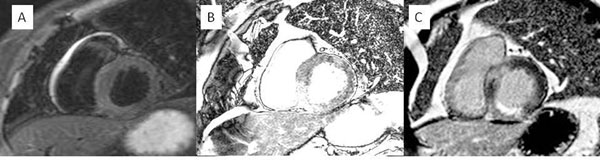
Image 2A) T2-STIR, B) ACUT2E and C) EGE.

